# Reduced Sensitivity to Negative Feedback May Lead to Risky Decision‐Making in Amphetamine Users

**DOI:** 10.1111/adb.70041

**Published:** 2025-05-05

**Authors:** Yu‐Hua Liu, Chiao‐Yun Chen, Neil G. Muggleton

**Affiliations:** ^1^ Department of Criminology National Chung Cheng University Chiayi Taiwan; ^2^ Cognitive Intelligence and Precision Healthcare Center National Central University Taoyuan Taiwan; ^3^ Institute of Cognitive Neuroscience National Central University Taoyuan Taiwan

**Keywords:** amphetamine, Balloon Analogue Risk Task (BART), Behavioural Inhibition System (BIS), ERN, FRN, risking decision making

## Abstract

In Taiwan, amphetamines are the main drug of abuse. While drug abuse is often related to individual risky decision‐making, how this relates to underlying neural mechanisms in amphetamine abusers remains unclear. The current study was carried out to help better understand this. A Balloon Analogue Risk Task (BART) was used to examine individual risky decision‐making in conjunction with event‐related potential (ERP) recording and presentation of questionnaires relating to behavioural control. Compared with healthy controls, amphetamine users had a lower score on the Behavioural Inhibition System (BIS) scale and showed reduced amplitudes in feedback‐related negativity (FRN) and error‐related negativity (ERN) ERP components following negative feedback on the task. Amphetamine users were less sensitive to punitive or aversive stimuli. This reduced sensitivity might lead to a higher tendency for risky decision‐making, with them less able to learn from mistakes and thus repeatedly engage in risky behaviours.

## Graphical abstract

Brain electrical activity while making risky decisions showed that amphetamine users had less sensitivity to punishment/aversive stimuli. This may underlie risky behavior and worse learning from mistakes in these individuals. It is possible that such factors contribute to their choice to use amphetamines.

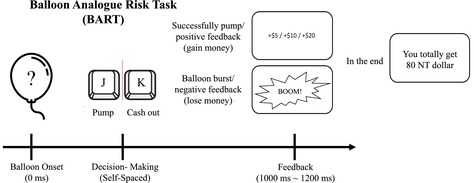

## 1 Introduction

Most countries in East and Southeast Asia, including China, Japan, South Korea, Thailand, and the Philippines, report amphetamines as their primary drug of concern [1]. In Taiwan, amphetamine users account for 30.7% of the official drug user population and 50.1% of the users cite drug dependence as the primary reason for use (with stress relief at 16.4% being the second most common reason) [2]. Amphetamine users have also been found to exhibit impulsive traits [3], and there has been shown to be a relationship between impulsive traits and risky behaviours [4]. With this trait, despite knowing that drug use not only harms their health but may also lead to legal issues, these users still choose to use amphetamines repeatedly. This tendency toward risky decision‐making is also believed to play a significant role in their relapse [5]. To better understand why these users continue to choose drug use despite negative consequences, this study investigated the nature of the impulsive traits and risky decision‐making among amphetamine users that may predispose them to making risky choices.

Ernst and Paulus [6] divided decision‐making into three phases based on time and functionality: assessment, execution and outcome processing. In the assessment phase, individuals receive different stimuli that predict measurable rewards or negative outcomes and form evaluations and preferences for each [6]. This phase involves brain regions such as the dorsal anterior cingulate cortex (dACC) [7] and dorsolateral prefrontal cortex (DLPFC) [8], commonly associated with functions like stimulus encoding and probability assessment. Previous study showed decision‐making deficits in amphetamine users were related to the dlPFC [9] and that stimulating the dlPFC effectively improved their decision‐making deficits and reduced cue‐induced craving [9, 10]. In addition, some brain regions related to the emotional circuitry, such as the amygdala and insula, are also believed to participate in the formation of preferences due to the emotional component conveyed by the stimuli [11]. During the execution phase, based on preference forming in the assessment phase, individuals suppress competing behaviours, plan the timing of their actions and ultimately ensure the successful execution of those actions [6], with this involving the engagement of DLPFC [12], the nucleus accumbens [13] and ACC [14]. Ultimately, in the outcome processing phase, individuals receive feedback on their actions and, similar to the first phase, engage in cognitive and emotional evaluation of this feedback [6]. However, unlike the first phase, where preferences are formed based on expected value, this phase evaluates the actual value resulting from the behaviour. In reinforcement learning (RL) theory, the differences between these are referred to as prediction errors (PEs) [15] and serve as a learning signal, prompting individuals to adjust their behaviour to become more adaptive [16, 17]. Some brain regions influenced by dopaminergic neurons, such as the ventral striatum [18] and the orbitofrontal cortex [18], are associated with the processing of PEs. Therefore, amphetamine users, who continue to engage in risky drug use despite experiencing negative feedback from their substance use, may have differences in how they process PEs in the outcome processing phase.

Reinforcement learning theory has been shown to be associated with the ERP components error‐related negativity (ERN) and feedback‐related negativity (FRN) [14]. ERN and FRN share many similarities and are both believed to originate from the ACC [19], suggesting that they stem from the same dopamine‐regulated system but are triggered in different situations. ERN is related to early unconscious attentional responses to errors [19], typically evoked from 50 to 150 ms. When individuals receive negative feedback, even if the they do not subjectively think that their decision caused the negative feedback, their brain may still recognise this decision‐making behaviour as a mistake and activate the error monitoring mechanism to adjust subsequent behaviour to avoid repeating such a mistake [20]. However, impulsive individuals tend to show a weaker ERN component [21]. Additionally, previous study showed that drug users had a reduced ERN amplitude, indicating a weaker ability to detect errors [22]. The other component, FRN, is an indicator of an individual's response to the expected outcome of a decision or action [23] and typically appears 250 to 300 ms after the onset of feedback [24]. It reflects the influence of dopamine signals on neurons in the ACC [25]. Generally, individuals expect positive feedback [26], and negative feedback is seen as an unexpected result, so this prediction error may induce higher FRN amplitude when negative feedback is given [23]. A previous study using a strategic gambling task observed that when individuals lost money, in addition to seeing increased FRN amplitudes, there was a higher likelihood of changing their original choice on the next decision stage [27]. However, Zhong et al. [28] found that amphetamine users showed a lower FRN amplitude to negative feedback, indicating a deficit in the feedback process in amphetamine users.

The P3 component, peaking at approximately between 300 and 600 ms after feedback onset [29] and generated by ACC and other frontal regions [30], has been related to stimulus evaluation, emotional rewarding and reward processing [30, 31]. Previous studies showed that task‐related stimuli, emotional stimuli and unexpected outcomes may all trigger a higher P3 amplitude [30]. Moreover, P3 is associated with the later stages of information processing and motivation [32]. In decision‐making, a larger P3 amplitude may indicate an increased craving for reward [33] and may also reflect an individual's motivation to participate in risky behaviours [30, 34]. Previous studies also found that individuals showed a larger P3 amplitude when facing loss ([31]；[35]). For instance, Kóbor et al. [35] used the BART to examine the risky decision making of a high executive function group and a low executive function group. They found that negative feedback (loss) triggered a higher P3 amplitude than the positive feedback (gain). Another study found heavy alcohol users had a significantly decreased P3 amplitude when receiving both negative and positive feedback, showing they may have a deficit in feedback processing.

Previous research has indicated that individuals with a more activated Behavioural Activation System (BAS) and a less activated Behavioural Inhibition System (BIS) (BAS+/BIS−) tended to exhibit impulsive traits, characterised by a strong pursuit of rewards and a diminished concern for potential negative consequences or punishments, a pattern also found to be associated with drug abuse [36] and risky decisions in a gambling task [37]. The BIS and BAS, derived from Reinforcement Sensitivity Theory (RST; [38]), have been found to relate to risky or safe decisions following reward or punishment feedback in gambling tasks [37], highlighting a correlation between BIS/BAS subscales and decision‐making. The BIS is said to be sensitive to punishment and aversive stimuli, so that individuals may inhibit some behaviours to avoid potential punishment or aversive stimuli, ultimately preventing individuals from engaging in behaviours with negative consequences [38]. On the other hand, the BAS is sensitive to reward and can be divided into three components: reward responsiveness, drive and fun‐seeking. Reward responsiveness reflects the individual's expectation and positive response to reward; drive represents the individual's persistence in goal‐oriented behaviour; fun‐seeking stands for engaging in seeking of exciting experiences [38]. However, some studies have shown inconsistent results, indicating no correlation between the BIS/BAS scales and decision‐making [39, 40]. Nevertheless, this study aimed to investigate whether amphetamine users' tendency to continue making risky decisions after receiving negative feedback was related to their personality traits and so the BIS/BAS scale was used for this.

To investigate the risky decision‐making of amphetamine users and examine whether feedback influenced their subsequent choices, this study used the Balloon Analogue Risk Task (BART), as used by Gu et al. [41]. In each trial of the BART, participants can pump a visually‐displayed balloon through multiple rounds. After each pump, one of two outcomes can occur: successful inflation, where the balloon expands (positive feedback), resulting in an increase in the accumulated monetary reward, or balloon explosion (negative feedback), where the accumulated monetary reward is lost. The accumulated reward and the level of economic risk varies with each decision on a trial—the further into the trial, the larger the accumulated reward and the greater the risk. This study expected to observe the same BAS+/BIS− impulsive personality traits in amphetamine users as in previous studies, which would be reflected in their behavioural performance, showing a higher risky ratio and shorter risky reaction time. Additionally, we expected that amphetamine users would have poorer processing of prediction errors during the feedback phase compared with the control group, leading to weaker ERN, FRN and P3 components during negative feedback.

## 2 Materials and Methods

### 2.1 Participants

The study was approved by the Ethics Committee of Kaohsiung Chang Gung Memorial Hospital, and all participants had a clear understanding of the content of the experiment and signed an informed consent form before taking part. The amphetamine users included in this study were all referred for mandatory treatment through deferred prosecution, and their drug history, according to official records, indicated they only used amphetamines. Thirty amphetamine users were recruited from hospital, based on a negative urine test, while 30 healthy controls without a history of drug use were recruited from the local community. All participants had normal or corrected‐to‐normal vision and had no history of mental illness or brain trauma. We excluded individuals who did not select the cash‐out option on the task and thus had no conservative reaction times (1 amphetamine user, 1 control participant), those with average reaction times below the minimum human reaction threshold (200 ms) (two amphetamine users) and those with extremes for their average reaction times (more than two standard deviations above the mean) (three amphetamine users, four control participants). In addition, three amphetamine users were excluded due to insufficient acceptance rates after preprocessing, and three amphetamine users and one control participant were excluded due to having fewer than 16 valid trials. Ultimately, 18 amphetamine users were included in the analysis, and, after matching for age and Raven's Progressive Matrices (RPM) scores [42] as closely as possible, 18 control participants were also included in the subsequent statistical analysis.

Thus, all participants in the experiment were right‐handed and included 18 amphetamine users (mean age = 36.83 years, standard error of mean [S.E.M.] = 2.29, 1 female) and 18 healthy controls (mean age = 31.00 years, S.E.M. = 2.25, 1 female). Amphetamine users in the study exclusively used amphetamines, with an average drug use duration of 16.4 years, and were deferred prosecution cases, referred to the hospital for addiction treatment as part of such deferral. Despite matching attempts, a significant RPM difference was seen between amphetamine users (mean = 10.00, S.E.M. = 1.26) and the healthy controls (mean = 19.56, S.E.M. = 1.17) (*F*_(1, 33)_=30.961, *p* < 0.001). Therefore, the analysis in the experiment included RPM scores as a covariate.

### 2.2 Procedure and BART

**FIGURE 1:**
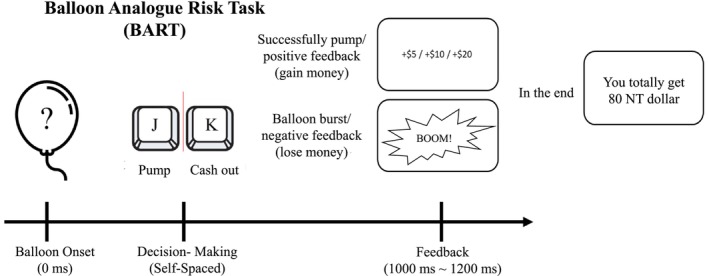
Balloon Analogue Risk Task. The participants could freely decide whether to pump the balloon. If the balloon was successfully pumped, the participants got a monetary reward. If the participants decided not to pump the balloon, they could choose to cash out the accumulated money for the current balloon and end the trial.

The participants first signed a consent form indicating their agreement to participate in the experiment. The BIS/BAS scales were used to measure individuals' personality [43]. The BART was presented on a laptop computer using the E‐Prime 3.0 software (Psychology Software Tools, Pittsburgh, PA, USA), and the design was similar to that of Gu et al. [41] with a total of 120 equally divided into five blocks of 24 trials. The distance between the participants and the computer screen was 50 cm. At the beginning of each trial, a balloon was presented in the centre of the screen, and the participants could freely decide whether to ‘pump it’. There were three rounds in each trial; that is, the maximum times that the balloon could be pumped was three, after which the next trial would be started. If the participants decided to pump the balloon, they carried this out by pressing the ‘F’ key on a computer keyboard. There were two possible results following a ‘pump’: if the balloon was successfully pumped, the participants got a monetary reward (positive feedback: 5 NT dollars for first round, 10 NT dollars for second round and 20 NT dollars for third round); if the balloon burst, all the money accumulated for the current balloon would be lost (negative feedback). If the participants decided not to pump the balloon, they could press ‘J’ to cash out the accumulated money in the current balloon and end the trial. The probability of a successful pump or a burst was equal (i.e. 50% success, 50% burst), but this was not told to the participants. This decision stage would continue until the participants made a choice of whether to pump or not. Feedback was then shown on the screen for 1000 to 1200 ms. After the final trial, the total accumulated money was also shown on the screen. See Figure 1 for an illustration of the task.

The following behavioural data from the task was analysed: (1) risky ratio for each round: the times of participants pumped divided by the total number of decisions; (2) risky reaction time for each round (the average time taken to make the ‘pump’ decision): the total time taken to pump divided by the total times the decision was made to pump; (3) the conservative reaction time for each round (the average reaction time of ‘cash‐out’ decision): the total time take for cash‐out decisions divided by the total times of cash‐out.

### 2.3 Electrophysiological Recordings and Statistical Analysis

Electroencephalography (EEG) data was collected using St.EEG VEGA with 32 channels of detachable gel‐free Ag/AgCl sensors (Artise Biomedical Co., Ltd, Hsinchu, Taiwan) with a sampling rate of 500 Hz, and the frequency range of the raw data was from 0 to 131 Hz. Electrode impedances were kept below 10 kΩ during data collection and electrodes A1 and A2 served as reference points. Additionally, an EOG removal model [44] was applied to the raw data for the whole experiment.

EEGLAB was used to preprocess EEG data. Data from all electrodes was subject to Artefact Subspace Reconstruction (ASR), and when data values were beyond 20 standard deviations of the mean amplitude in a 0.5‐s window, the data was rejected. In addition, a 6‐dB band pass filter with a high‐pass of 0.5 Hz and a low‐pass of 30 Hz was used, and the resulting data were segmented with time locked from 200 ms pre‐stimulus to 800 ms post‐stimulus. These epochs were baseline corrected relative to the −200‐ to 0‐ms period and averaged for the successful pump (positive feedback) and the balloon burst (negative feedback) trials respectively. The time of successful inflation or of balloon burst were used as the onset time for the decision‐making related components at the electrode sites of Fz, FCz and Cz:FRN and P3. The time window of the FRN component was defined as 160 to 260 ms and for the P3 component was defined as 300 to 400 ms. The ERN component used the balloon burst time as the onset at the electrode sites of Fz, FCz and Cz and the time window was defined as 90 to 150 ms.

SPSS 26.0 was used to carry out the data analysis, and a *p* value < 0.05 was considered significant. As mentioned above, because the RPM scores showed a significant difference between the two groups, RPM scores were included as a covariate in the main analysis. Next, one‐way analysis of variance (ANOVA) was performed to test for any group differences for behavioural performance and for ERP mean amplitudes.

## 3 Results

### 3.1 Questionnaire Results

See Table 1 for the questionnaire results. There was a significantly lower BIS score in the amphetamine users than in the healthy controls (*F*_(1, 33)_ = 45.562, *p* < 0.001, *η* ^2^ = 0.580). No other significant group differences were seen in all BAS subscales (BAS‐drive:*F*_(1, 33)_ = 0.531, *p* = 0.471, *η* ^2^_*p*_ = 0.016; BAS‐fun seeking: *F*_(1, 33)_ = 1.462, *p* = 0.235, *η* ^2^_*p*_ = 0.042; BAS‐reward response: *F*_(1, 33)_ = 3.621, *p* = 0.066, *η* ^2^_*p*_ = 0.099).

**TABLE 1:** Questionnaire results for amphetamine users and control participants.

Questionnaire score		Amphetamine users (*n* = 18)		Controls (*n* = 18)		*p*
		M	SD	M	SD	
BAS^a^	Reward responsiveness	15.67	4.49	19.22	2.80	0.066
	Drive	11.56	4.03	13.17	2.75	0.471
	Fun seeking	10.11	3.91	12.89	2.17	0.235
BIS^b^		17.61	3.53	23.28	2.97	<0.001**^c^

^a^ BAS: Behavioural Activation System.

^b^ BIS: Behavioural Inhibition System.

^c^ *p*‐values: * indicates significance at *p* < 0.05 and ** indicates significance at *p* < 0.01.

### 3.2 Behavioural Results

There was no significant group difference in risky ratio (first round: *F*_(1, 33)_ = 3.525, *p* = 0.069, *η* ^2^_*p*_ = 0.097; second round: *F*_(1, 33)_ = 0.216, *p* = 0.645, *η* ^2^_*p*_ = 0.007; third round: *F*_(1, 33)_ = 0.807, *p* = 0.375, *η* ^2^_*p*_ = 0.024), risky reaction time (first round: *F*_(1, 33)_ = 1.202, *p* = 0.281, *η* ^2^_*p*_ = 0.035; second round: *F*_(1, 33)_ = 2.731, *p* = 0.108, *η* ^2^_*p*_ = 0.076; third round: *F*_(1, 33)_ = 1.133, *p* = 0.295, *η* ^2^_*p*_ = 0.033) and conservative reaction time (first round: *F*_(1, 33)_ = 0.032, *p* = 0.858, *η* ^2^_*p*_ = 0.001; second round: *F*_(1, 33)_ = 0.245, *p* = 0.624, *η* ^2^_*p*_ = 0.007; the third round: *F*_(1, 33)_ = 0.687, *p* = 0.413, *η* ^2^_*p*_ = 0.020) (Table 2).

**TABLE 2:** Behavioural results for amphetamine users and control participants.

BART		Amphetamine users (*n* = 18)		Controls (*n* = 18)		*p*
		M	SD	M	SD	
Risky ratio	R1^a^	0.83	0.17	0.93	0.08	0.069
	R2^b^	0.79	0.19	0.76	0.16	0.645
	R3^c^	0.60	0.30	0.41	0.18	0.375
Risky reaction time	R1	690.81	269.98	612.45	263.84	0.281
	R2	847.52	363.34	712.00	285.60	0.108
	R3	1044.44	530.09	889.08	603.62	0.295
Conservative reaction time	R1	802.92	463.00	611.64	447.31	0.858
	R2	875.09	478.45	954.48	535.88	0.624
	R3	822.10	556.38	769.29	393.19	0.413

^a^ R1: Round 1.

^b^ R2: Round 2.

^c^ R3: Round 3.

### 3.3 ERP Results

**FIGURE 2:**
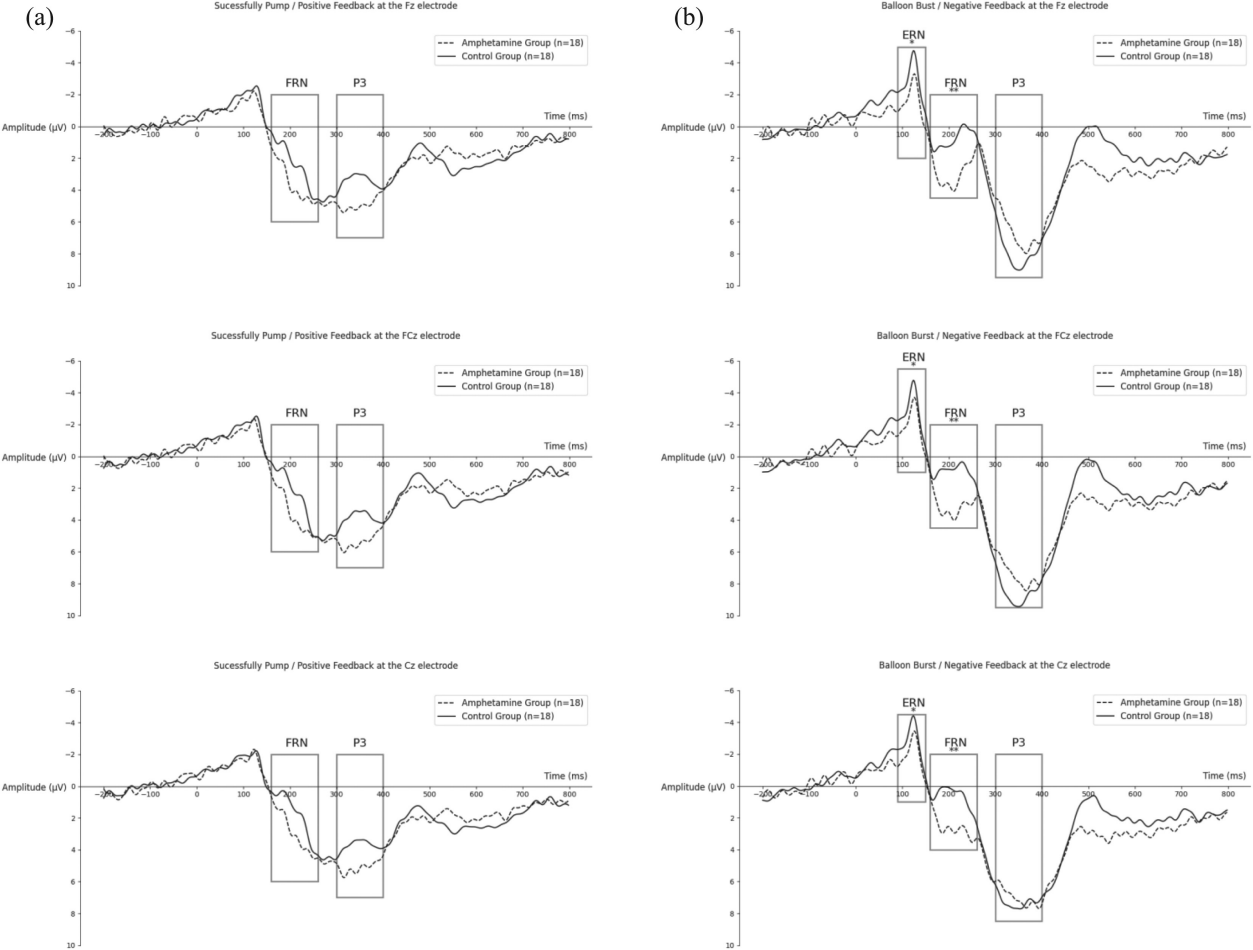
ERP grand averages recording BART performance. Data are shown for (a) the successfully pump/positive feedback and (b) for the balloon burst/negative feedback.

The mean amplitudes of the FRN and the P3 at Fz, FCz and Cz were calculated for the positive feedback (Figure 2a). *F*‐tests showed that there were no significant group differences in FRN (Fz: *F*_(1, 33)_ = 0.201, *p* = 0.656, *η* ^2^_p_ = 0.006; FCz: *F*_(1, 33)_ = 0.560, *p* = 0.460, *η* ^2^_*p*_ = 0.017, Cz: *F*_(1, 33)_ = 0.971, *p* = 0.332, *η* ^2^_*p*_ = 0.029) or P3 (Fz: *F*_(1, 33)_ = 1.991, *p* = 0.168, *η* ^2^_*p*_ = 0.057; FCz: *F*_(1, 33)_ = 3.186, *p* = 0.083, *η* ^2^_*p*_ = 0.088; Cz: *F*_(1, 33)_ = 2.731, *p* = 0.108, *η* ^2^_*p*_ = 0.076).

The mean amplitudes of the ERN, the FRN and the P3 at Fz, FCz and Cz were calculated for the negative feedback (Figure 2b). ERN (Fz: *F*_(1, 33)_ = 6.196, *p* = 0.018, *η* ^2^_*p*_ = 0.158; FCz: *F*_(1, 33)_ = 4.869, *p* = 0.03, *η* ^2^_*p*_ = 0.129, Cz: *F*_(1, 33)_ = 4.296, *p* = 0.046, *η* ^2^_*p*_ = 0.115) and FRN (Fz: *F*_(1, 33)_ = 5.980, *p* = 0.020, *η* ^2^_*p*_ = 0.153; FCz: *F*_(1, 33)_ = 8.473, *p* = 0.007, *η* ^2^_*p*_ = 0.204, Cz: *F*_(1, 33)_ = 11.227, *p* = 0.002, *η* ^2^_*p*_ = 0.254) both reached significance, showing the amphetamine users had significantly smaller negative feedback ERN amplitudes and FRN amplitudes. P3 was not significantly different (Fz: *F*_(1, 33)_ = 0.002, *p* = 0.966, *η* ^2^_*p*_ = 0.00; FCz: *F*_(1, 33)_ = 0.067, *p* = 0.798, *η* ^2^_*p*_ = 0.002, Cz: *F*_(1, 33)_ = 0.383, *p* = 0.540, *η* ^2^_*p*_ = 0.011).

## 4 Discussion

This study utilised the BART to investigate risky decision tendencies and feedback processing in amphetamine users and found two main results. First, contrary to our hypothesis, amphetamine users only exhibited lower BIS scores. BIS is related to sensitivity to punishment and aversive stimuli, and it is associated with the inhibition of behaviours that lead to negative consequences. The lower BIS scores may reflect weaker sensitivity to punishment and aversive stimuli in amphetamine users, which impairs their ability to inhibit behaviours that result in negative outcomes. Considering that the average drug use duration among our recruited participants was 16.4 years, long‐term drug use may desensitise their reward system to rewards, leading to a weakened response to anticipated rewards. As a result, they may not exhibit strong BAS traits and may even show a tendency toward weaker BAS‐Reward Responsiveness.

To eliminate potential gender differences introduced by one female participant, we excluded the female participants and reanalysed the remaining male participants to verify the results of our current study. We discovered two new findings: (1) BAS‐Reward Responsiveness: amphetamine users scored significantly lower than controls. (2) Risky Ratio in R1: amphetamine users exhibited a lower risky ratio in the first round. The other results were consistent with the analysis that included females. For these two new findings, we believe that long‐term drug use has led to a reduced response in the reward system among male amphetamine users, producing the BAS (Reward Responsiveness) ‐ /BIS ‐ pattern. Furthermore, the lower initial risky ratio may correspond to the lower BAS reward responsiveness characteristics observed in the questionnaire, suggesting that male amphetamine users have lower expectations for monetary rewards compared with controls, which in turn decreases their motivation for balloon inflation.

Consistent with the low BIS score, amphetamine users showed significantly smaller amplitudes for both ERN and FRN ERP components than controls. In general, individuals expect positive feedback, so the PEs between the actual negative feedback and the expected positive outcome should serve as learning signals, prompting individuals to adjust their choice [16, 17]. Moreover, decisions that caused the negative feedback will be recognised as a mistake and activate the subsequent error monitoring mechanism [20]. However, the smaller ERN when faced with a balloon burst may indicate amphetamine users had a lower sensitivity to risky responses resulting in negative feedback (error responses). Previous research has shown that feedback processing deficits in substance users are associated with impaired impulse control and error monitoring mechanisms [22]. Such weak error sensitivity may mean amphetamine users have lower activation of the error monitoring mechanisms in the brain, leading to increased likelihood of make the same, erroneous decision again. Another study indicated that individuals using stimulants exhibited abnormal activation patterns in the left insula and bilateral DLPFC, leading to poorer adaptation to error frequency [45]. This resulted in the deployment of fewer brain resources during decision‐making processes, making it difficult to predict error performance.

Moreover, a smaller FRN also showed that when facing a loss of money or unexpected outcomes amphetamine users had a lower degree of response, showing a lower sensitivity to negative feedback. This low sensitivity to negative feedback may cause amphetamine users fail to activate a subsequent behaviour adjustment after receiving such feedback. Because ERN and FRN both originate from the ACC and are thought to stem from the same dopaminergic system [19], reduced ERN and FRN may suggest that the dopaminergic system in the ACC was disrupted by long‐term drug use, leading to the decreased sensitivity to both errors and negative feedback. These changes in reward dopaminergic system may involve alterations in the prefrontal cortex and nucleus accumbens and were linked to reduced sensitivity to punishment and increased tendency for risk‐taking behaviour [46]. Additionally, study on stimulant users also found difficulty in learning from negative feedback [47]. This loss of sensitivity may indicate that amphetamine users were unable to effectively learn through PEs and initiate error monitoring and subsequent behavioural adjustments and could lead them to persist in making high‐risk, erroneous choices.

Although this study did not find significant behavioural results, we still observed that both groups exhibited lower risky ratios as the potential loss amount increased. This aligns with prospect theory, which suggests that people make decisions by mentally setting a reference point and evaluating how the outcomes of their decisions differ from it [48]. Most individuals tend to be much more sensitive to losses than to gains, a phenomenon known as loss aversion [48]. In the BART, participants may view the accumulated monetary amount as a reference point. Thus, even though risky decisions could potentially lead to higher gains, the amplified perception of potential losses may lead to individual avoiding risks. Interestingly, the control group's risky ratio dropped from 93% to 41% (52% decrease), while the amphetamine users' risky ratio only decreased from 83% to 60% (23% decrease). The decreasing trend in the risky ratio among amphetamine users, despite increasing risk levels, may indirectly reflect their lower sensitivity to negative feedback. This suggested that their diminished response to negative outcomes could contribute to a tendency to persist in risk‐taking decisions.

While the ERP results seem inconsistent with the absence of behavioural effects, some reasons may account for this. For example, there may be differences in sensitivity and distinct underlying neural mechanisms for the two types of effect. ERPs, such as ERN and FRN, are more sensitive in detecting subtle cognitive and neural differences, while behavioural measures provide a broader view of performance. Previous studies, like that of Fukushima and Hiraki [49], also found inconsistencies between ERP results and behavioural outcomes, with significant ERP differences under different conditions despite the absence of behavioural differences. Moreover, even when similar behaviours are observed, they may not reflect identical neural processes. In individuals with substance use disorders, compensatory mechanisms may help maintain performance despite neural processing deficits [50]. This could explain why amphetamine users in our study showed reduced sensitivity to negative feedback in ERPs but no significant behavioural differences. Future research could explore these compensatory strategies using neuroimaging or more complex decision‐making tasks.

This study has some limitations, which should be noted. First, due to the small sample size, caution should be employed when considering generalisation of the findings here to amphetamine users in general. Future work testing more participants could help determine if these findings can be replicated and so increase confidence in such a generalisation. Secondly, due to recruitment difficulties and exclusions during analysis, this study ultimately included only one female participant, which may have led to gender imbalance in the results. Future studies should aim to recruit a more balanced sample in terms of gender to better explore potential sex differences and ensure the generalisability of the findings. Moreover, the current study design set the balloon explosion probability at 50% without informing the participants of this likelihood. As a result, we were unable to examine whether amphetamine users exhibit reduced sensitivity to negative feedback in high‐ or low‐risk situations. Future research could build on these findings by dividing the BART task into high‐risk condition (higher explosion probability) and low‐risk condition (lower explosion probability) to investigate whether amphetamine users' risk‐taking tendencies and sensitivity to negative feedback differ across varying risk conditions. Another limitation of using a fixed 50% explosion probability in the BART was that this means the task does not fully capture the complexity and uncertainty of real‐world risk‐taking scenarios. Future studies could address this limitation by incorporating adaptive probability structures or varying risk levels to better simulate real‐world decision‐making processes.

## 5 Conclusions

ERP data collected from amphetamine users showed effects consistent with questionnaire data, with both showing amphetamine users had weaker sensitivity to negative feedback/punishment/errors. This indicates that amphetamine users may not be able to effectively respond and adapt to negative feedback, and so have more difficulty modifying or adjusting their behaviour correctly. This may be a contributing factor to amphetamine users having difficulties in quitting drug use, with amphetamine users failing to learn effectively from either punishment or negative feedback to adjust their decision‐making behaviour, and thus persist with the ‘risky decision’ of continued drug use.

## Author Contributions

All authors contributed to the design of the study. Y.‐H.L. collected the data and carried out initial manuscript preparation which was then refined by C.‐Y.C. and N.G.M. C.‐Y.C. supervised the study. All the authors read and approved the final manuscript.

## Ethics Statement

The study was approved by the Ethics Committee of Kaohsiung Chang Gung Memorial Hospital, and all participants had a clear understanding of the content of the experiment and signed an informed consent form before taking part.

## Conflicts of Interest

The authors declare no conflicts of interest.

## Data Availability

Data are available on request from the authors.
